# A Plurizyme with Transaminase and Hydrolase Activity Catalyzes Cascade Reactions

**DOI:** 10.1002/anie.202207344

**Published:** 2022-08-04

**Authors:** Sergi Roda, Laura Fernandez‐Lopez, Marius Benedens, Alexander Bollinger, Stephan Thies, Julia Schumacher, Cristina Coscolín, Masoud Kazemi, Gerard Santiago, Christoph G. W. Gertzen, Jose L. Gonzalez‐Alfonso, Francisco J. Plou, Karl‐Erich Jaeger, Sander H. J. Smits, Manuel Ferrer, Víctor Guallar

**Affiliations:** ^1^ Department of Life Sciences Barcelona Supercomputing Center Carrer de Jordi Girona, 31 08034 Barcelona Spain; ^2^ Department of Applied Biocatalysis, ICP, CSIC Marie Curie 2 28049 Madrid Spain; ^3^ Center for Structural Studies Heinrich-Heine-University, Building 26.44.01.62 Universitaetsstr 1 40228 Duesseldorf Germany; ^4^ Institute of Molecular Enzyme Technology Heinrich-Heine-Universität Düsseldorf, Building 26.44.01.62 Universitaetsstr 1 40228 Duesseldorf Germany; ^5^ Forschungszentrum Jülich, Building 15.8, 01/303, 52428 Wilhelm Johnen Straße Jülich Germany; ^6^ Institució Catalana de Recerca i Estudis Avançats Passeig de Lluís Companys, 23 08010 Barcelona Spain

**Keywords:** Cascade Reactions, Computational Engineering, Esterase, Plurizyme, Transaminase

## Abstract

Engineering dual‐function single polypeptide catalysts with two abiotic or biotic catalytic entities (or combinations of both) supporting cascade reactions is becoming an important area of enzyme engineering and catalysis. Herein we present the development of a *PluriZyme*, TR_2_E_2_, with efficient native transaminase (*k*_cat_: 69.49±1.77 min^−1^) and artificial esterase (*k*_cat_: 3908–0.41 min^−1^) activities integrated into a single scaffold, and evaluate its utility in a cascade reaction. TR_2_E_2_ (pH_opt_: 8.0–9.5; *T*_opt_: 60–65 °C) efficiently converts methyl 3‐oxo‐4‐(2,4,5‐trifluorophenyl)butanoate into 3‐(*R*)‐amino‐4‐(2,4,5‐trifluorophenyl)butanoic acid, a crucial intermediate for the synthesis of antidiabetic drugs. The reaction proceeds through the conversion of the β‐keto ester into the β‐keto acid at the hydrolytic site and subsequently into the β‐amino acid (e.e. >99 %) at the transaminase site. The catalytic power of the TR_2_E_2_ *PluriZyme* was proven with a set of β‐keto esters, demonstrating the potential of such designs to address bioinspired cascade reactions.

## Graphical abstract

A *PluriZyme* with transaminase and esterase activities was constructed by combining computational and laboratory design methods. The enzyme can perform cascade reactions in a single protein scaffold. Experimental evidence is presented for the catalytic efficiency of both the native transaminase and the artificial esterase active site and their synergistic action to transform β‐keto esters into enantiopure β‐amino acids.

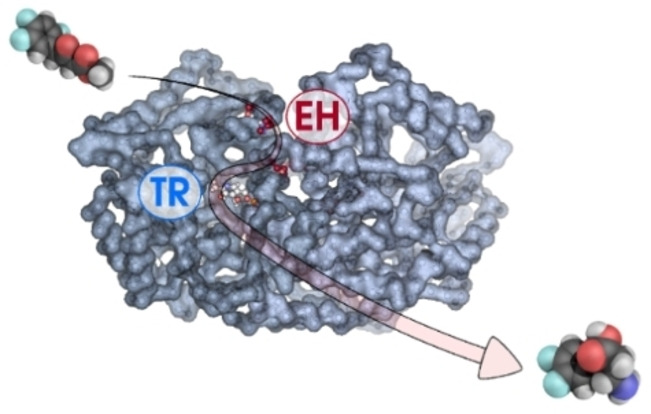

## Introduction

Microorganisms are regarded as self‐replicating containers of enzymes that catalyze enzyme cascades (or biosynthetic pathways), yielding complex products.^[1]^ Cascade reactions are also relevant in the field of biocatalysis for the synthesis and assembly of numerous molecules.^[2]^ In vitro cascades have been investigated with at least two enzymatic reactions combined with a chemical step^[3]^ or purely enzymatic systems, excluding enzymes required for the regeneration of cofactors or the removal of poisonous side products.^[2]^ Thus, multienzyme systems and biomimetic or bioinspired architectures have been built to spatially organize different enzymes with nanometer precision.[^4^, ^5^, ^6^] Using this approach, the transport of intermediates between enzymes in close proximity, known as proximity channeling, is efficiently controlled, thus favoring the progression of chain reactions.[^7^, ^8^] Recent examples illustrating the design of microfluidic electrospray microcapsules mimicking natural subcellular compartments, such as organelles or organs,^[9]^ or onion‐like photonic spheres,^[10]^ demonstrate the relevance of systems that minimize the diffusion of intermediates between enzymes, enhancing the overall efficiency of bioreactions. At the nanoscale, artificial chimeric proteins in which two modules, domains, or enzymes, each being different concerning the chemistry or reaction step catalyzed, are joined via a linker have also been developed. An example is the design of enzyme chimeras in which an endoglucanase and a β‐glucosidase are linked to support polysaccharide degradation.^[11]^ Other examples include the design of two‐enzyme polyethylene terephthalate depolymerization systems for plastic upcycling^[12]^ and the fusion of enzymes integrating terpene synthases.^[13]^

**Scheme 1:**
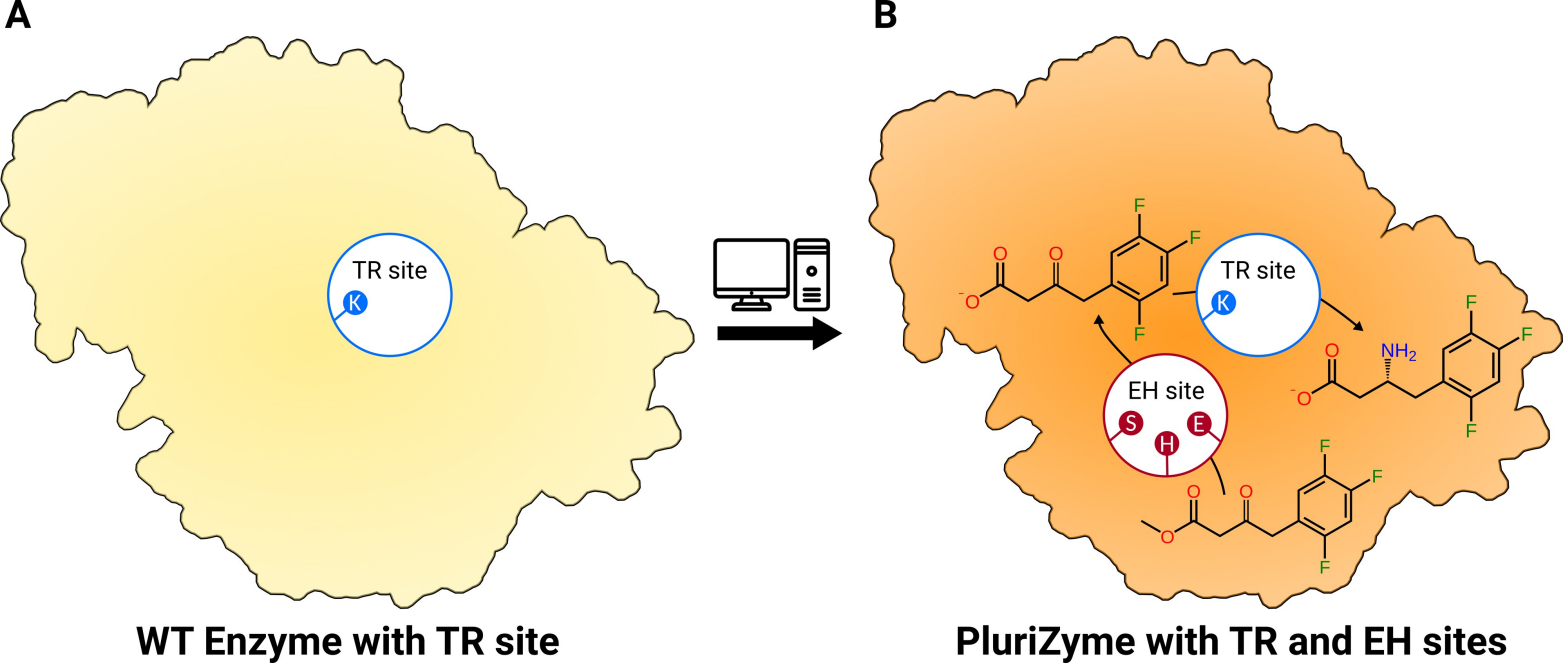
The general concept for transforming a wild‐type class III ω‐TA (A) into an artificial *PluriZyme* with transaminase (TR) and ester hydrolase (EH) activities (B). The cascade reaction supported by TR_2_E_2_, namely, the hydrolysis of 3‐OTfBE to methyl 3‐oxo‐4‐(2,4,5‐trifluorophenyl)butanoic acid (3‐OTfBA) and its further transamination to (*R*)‐3‐ATfBA, is shown. Methyl 3‐amino‐4‐(2,4,5‐trifluorophenyl)butanoate (3‐ATfBE) was detected as a minor by‐product.

The design of dual‐function catalysts characterized by the precise positioning of two abiotic^[14]^ or biotic^[15]^ catalytic entities (or a combination of both),^[16]^ that act in concert to catalyze synergetic reactions, represent complementary alternatives to the traditional two‐catalysts systems (either mixed or fused[^11^, ^12^, ^13^]). They have provided new solutions for activating and transforming specific molecules involving several steps, or even new‐to‐nature reactions.[^14^, ^15^, ^16^] The challenge originates from the difficulty in precisely controlling the positioning of the catalytic entities in a single protein scaffold, which is different from combining the active centers of two covalently bound enzymes.[^11^, ^12^, ^13^] The catalytic potential of an artificial protein scaffold with two abiotic catalytic groups has been recently demonstrated by the design of a chimeric streptavidin with two adjacent Au^I^ complexes structurally equal. Both gold entities can work individually but, through adopting multiple poses, can work in synergy to activate an alkyne.[^14^, ^17^] In a recent elegant study, the incorporation of a serine close to the haem cofactor of a P450 enzyme allowed the design of a dual‐function catalyst for efficient enantioselective carbene C−H insertion reactions.^[15]^ The serine residue contributes to controlling the orientation and activation of the reaction intermediates. Other examples of artificial protein scaffolds with combined abiotic and biotic catalytic groups supporting tandem catalysis have been reported.^[16]^ These recent studies exemplify the interest in designing multi‐function enzymes for synthetic chemistry. In this study, we went one step further and designed an enzyme with two biotic sites, one supporting hydrolase and one transaminase activity, that are independent catalytic entities but can also work in synergy (Scheme 1).

For this purpose, we used recently published computational tools to design *PluriZymes*,[^18^, ^19^] the name we assigned to the concept of adding multiple active sites to the same enzyme structure. In detail, we report the addition of a catalytic triad (Ser, His, Asp/Glu) and an oxyanion hole supporting ester hydrolysis in a class III ω‐transaminase (ω‐TA), which is subsequently referred to as TR_2_ (GenBank acc. nr. MH588437).^[20]^ ω‐TA is a well‐established enzyme used to design artificial biocatalytic linear cascades to prepare organic molecules,^[21]^ and thus, it was targeted in the present study. We designed a *PluriZyme*, hereafter referred to as TR_2_E_2_, able to catalyze the one‐pot cascade synthesis of the β‐amino acid (*R*)‐3‐amino‐4‐(2,4,5‐trifluorophenyl)butanoic acid (3‐ATfBA), a key precursor in the synthesis of gliptins (Scheme 1).[^22^, ^23^] Our results show that the design of this catalytically efficient *PluriZyme* is feasible and facilitates the generation of 3‐ATfBA from the methyl 3‐oxo‐4‐(2,4,5‐trifluorophenyl)butanoate (3‐OTfBE) β‐keto ester with an excellent conversion rate (>99 %; conc.: 14 mM) and enantioselectivity (e.e. >99 %). Moreover, by using a range of β‐keto esters, we further demonstrated that dual function *PluriZymes* are powerful catalysts for tandem reactions. In addition, we report the crystal structures of the TR_2_ enzyme and the newly designed TR_2_E_2_ *PluriZyme*.

## Results and Discussion

In this study, we targeted TR_2_, a class III ω‐TA (GenBank acc. nr. MH588437) isolated from the beach acidic pool on Vulcano Island and most likely derived from a bacterium of the *Acidihalobacter* genus.^[20]^ TR_2_ showed maximal transaminase activity at 45–55 °C, suggesting that it is moderately thermostable. It also showed a broad substrate range and efficiently converted bulky ketones and amines with (*R*) stereochemistry, which is rare among class III ω‐TAs.

We aimed to crystalize and solve the structure of TR_2_. We used sitting drop vapor diffusion and tested 2000 conditions from commercially available screens to achieve this goal. The obtained crystals were optimized in 24‐well sitting drop crystallization plates. The crystals of TR_2_ diffracted moderately, and a dataset was collected at 3.6 Å resolution (statistics for the data are presented in Table S1). The TR_2_ crystals were phased by molecular replacement using PHASER in the Phenix program,^[24]^ and a model calculated with AlphaFold as a template.^[25]^ The structure was then refined in iterative cycles of manual building and refinement in Coot, followed by software‐based refinements using the Phenix program suite.[^26^, ^27^]

**Figure 1:**
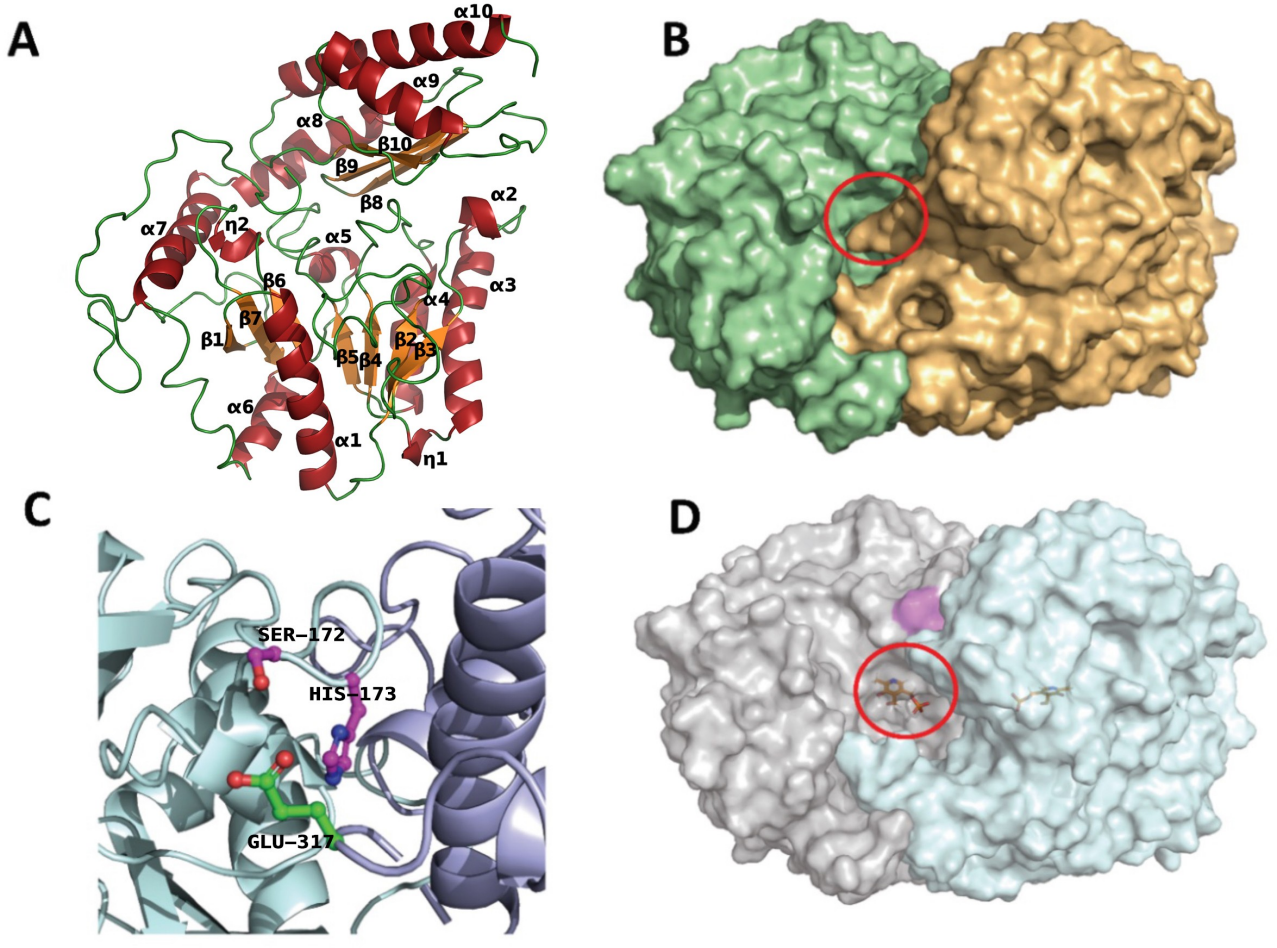
A) Secondary structural elements of TR_2_, with α‐helices (red), β‐strands (orange) and loops (green) shown. B) Surface representation of the TR_2_ dimer with highlighted transaminase binding site (red circle). C) Higher‐magnification image of the TR_2_E_2_ structure at the Ser172 and His173 mutations. D) Surface representation of the TR_2_E_2_‐PLP dimer with a highlighted transaminase binding site (red circle). PLP is located in the transaminase binding site and is shown as a ball‐and‐stick representation. The positions of the Ser172 and His173 mutations are colored magenta. The figure was created using PyMOL Version 2.3.2.

TR_2_ shows an asymmetric unit of four peptide chains building a functional dimer (Figure 1A, B). It presents the typical type I fold of class III ω‐TA observed in known structures present in the PDB database (Table S1),^[28]^ and it contains the characteristic pyridoxal‐5′‐phosphate (PLP)‐binding domain composed of β‐sheets and the catalytic base Lys289. The TR_2_ monomer is subdivided into a core domain, which is the PLP‐binding domain, and a C‐terminal subdomain. The core domain is composed of a central β‐sheet consisting of the strands β1–β7 surrounded by helices α1–α2 and the short helical structures η1 and η2. The smaller subdomain at the C‐terminus consists of the three α‐helices and β‐strands 8, 9, and 10. TR_2_ is a functional dimer, as represented by Figure 1B; here, the tunnel for accessing the binding pocket with the catalytic base (red circle) is composed of the two monomers (highlighted in different colors).

The protocol used to design an artificial hydrolase site has been explained previously,[^18^, ^19^] including two review articles.[^29^, ^30^] Briefly, the process begins with scanning the transaminase surface to identify noncatalytic ester binding sites using global Protein Energy Landscape Exploration (PELE) exploration.^[31]^ Next, we perform local explorations of active site variants to introduce a well‐positioned catalytic triad while also considering the existence of oxyanion holes that are required for efficient ester hydrolysis.^[32]^ Finally, optimum variants designed with PELE are ranked through i) Molecular Dynamics (MD) refinement simulations, accounting for the integrity of the triad, and ii) ΔΔ*G* estimations using the HotSpot Wizard.

**Figure 2:**
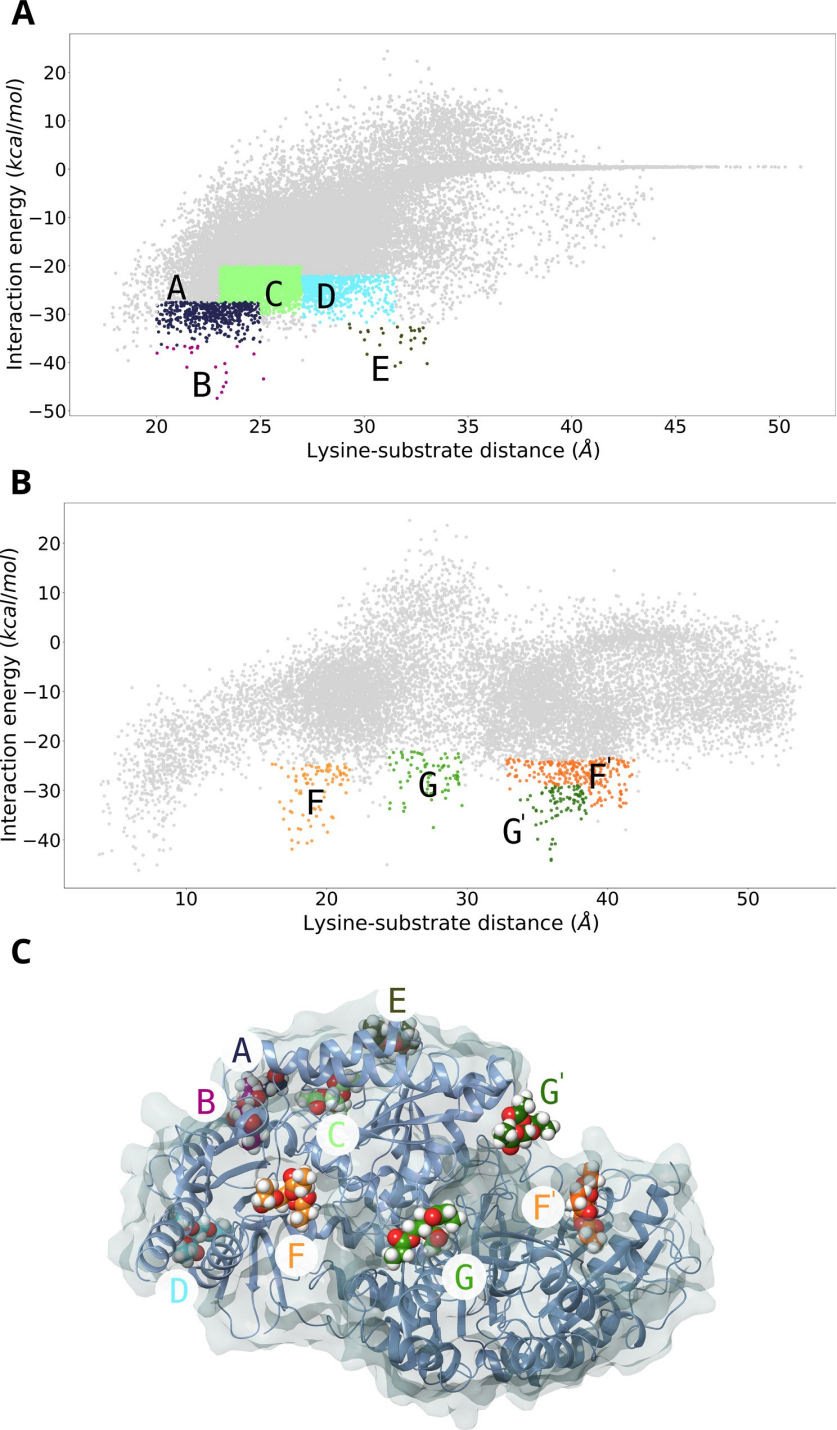
Global exploration of noncatalytic ester binding sites on the TR_2_ surface with PELE. Energetic profiles for the monomer (A) and dimer (B) are shown. Representative PELE steps around the noncatalytic ester binding sites are highlighted in the energetic profiles with a particular color (site A in dark blue, site B in purple, site C in lime, site D in turquoise, site E in dark green, sites F/F′ in orange for chains A and B and sites G/G′ in green for chains A and B). C) 3D structure of TR_2_ with representative binding poses of the probe ester in the different noncatalytic binding orientations. The surface is displayed in a transparent blue‐white color, and the secondary structure of each monomer is shown as ribbons. Substrate molecules highlight C atoms using the energetic profile colors (underlying the noncatalytic ester binding sites). Energetic profiles were created with the Matplotlib library.

As shown in Figure 2, several potential sites for ester binding were located with global exploration. During the local exploration, we focused on the enzyme‐substrate interaction energy (see Figure S1), the catalytic triad hydrogen bond lengths (see Figures S2–S5), and the occurrence (density) of catalytic poses (see Table S2) to introduce the catalytic triad in each of the sites described above. Typically, we aimed for the ester carbon to be located within 3–4 Å from the serine nucleophilic oxygen and catalytic hydrogen bonds to be located within reasonable distances (3.5 Å). These distance parameters, the root mean square deviation (RMSD) of the overall structure, and the local RMSD of the main catalytic lysine and the designed triad (see Figures S6–S8) were estimated from MD simulations. Then, a score ranking of all mutants was created from all these metrics and combined with the ΔΔ*G* stability calculation (see Table S3); the average of each metric was compared to ideal values obtained from the simulations to obtain the score. Although, the ΔΔ*G* stability calculation of the variants was just used to penalize or even discard the high destabilizing mutants (ΔΔ*G*>5 kcal mol^−1^), it has been proven to be crucial for enzyme design.[^33^, ^34^, ^35^] The final list contained 20 ranked mutants (only 6 were discarded due to drastic deviations in the metrics).

TR_2_ and the 20 designed mutants (Table S4) were produced and purified, and their transaminase activity was quantified to evaluate whether any of the mutations were deleterious to the original transaminase activity. Of the 20 mutants examined, only six showed transaminase activity, retaining 58 to 92 % of the original TR_2_ activity (*k*_cat_: 83.38±2.52 min^−1^) when using hexanal, a model aldehyde that was shown to be one of the preferred substrates for TR_2_ (see Table S4, Figure S9).^[20]^ These mutants contain from 1 to 3 mutations (Table S4) located at sites B, E, F, and G, as shown in Figure 2C.

All six mutants retaining transaminase activity were further examined for the presence of hydrolytic activity using model esters *p*‐nitrophenyl acetate (*p*NPA), propionate (*p*NPP), and butyrate (*p*NPB). As shown in Table 1, only purified TR_2_E_2_ (Figure S10), which retained approximately 80 % of the original transaminase activity (*k*_cat_: 69.48±1.77 min^−1^), was capable of hydrolyzing *p*NPA, *p*NPP, and *p*NPB, with the latter being the preferred substrate (*k*_cat_: 0.41±0.05 min^−1^). This variant contains two mutations, A172S and Q173H. We further observed that TR_2_E_2_ hydrolyzed 52 of a set of 96 chemically and structurally diverse esters tested (*k*_cat_: 3908 to 1.2 min^−1^) (Table S5). Under the same conditions, the activity for TR_2_ was below the detection limit for all esters. When the residues Ser172 and His173 were replaced with Ala, the activity was completely abolished. Thus, the A172S and Q173H mutations in TR_2_E_2_ (located at site G in Figure 2) do not exert a deleterious effect on the original transaminase activity and, importantly, confer esterase activity.

**Table 1:** Kinetic constants for the enzymatic activities of TR_2_E_2_.

Substrate	*K*_m_ [mM]	*k*_cat_ [min^−1^]	*k*_cat_/*K*_m_ [s^−1^ M^−1^]
Hydrolytic activity^[a,b]^			
*p*NPA^[a]^	2.056±0.239	0.323±0.010	2.62±0.70
*p*NPP^[a]^	2.664±0.642	0.409±0.051	2.57±1.3
*p*NPB^[a]^	0.279±0.096	0.112±0.010	6.67±1.75
3‐OTfBE^[b]^	2.407±0.038	32.47±1.13	224.9±11.4
3‐ATfBE^[b]^	4.750±0.503	0.131±0.02	0.460±0.12

Transaminase activity^[c]^			
3‐OTfBE^[c]^	12.27±0.47	0.771±0.20	1.05±0.07
3‐OTfBA^[c]^	7.65±0.35	92.91±1.16	202.5±11.8

Reaction conditions as detailed in Supporting Experimental Procedures: [a] *K*_m_‐[protein]: 4.5 μg mL^−1^; [ester]: 0–7 mM; reaction volume: 200 μL; *T*: 30 °C; pH: 7.0 (40 mM 4‐(2‐hydroxyethyl)‐1‐piperazineethanesulfonic acid (HEPES) buffer). [b] *K*_m_‐[protein]: 4.5 μg mL^−1^; [ester]: 0–25 mM; reaction volume: 44 μL; *T*: 30 °C; pH: 8.0 (5 mM 4‐(2‐hydroxyethyl)‐1‐piperazinepropanesulfonic acid (EPPS) buffer, plus 0.45 Phenol Red®). [c] *K*_m_‐[protein]: 4.5 μg mL^−1^; [substrate]: 0–100 mM; [PLP]: 1 mM; [2‐(4‐nitrophenyl)ethan‐1‐amine (NPEA)]: 0–100 mM; reaction volume: 200 μl; *T*: 40 °C; pH: 7.5 (100 mM K_2_HPO_4_ buffer). In all cases, *k*_cat_ was calculated assuming *v*=*k*_cat_ when *v*=μM product min^−1^ μM^−1^ enzyme. Raw data for the kinetic experiments are shown in Figure S11.

**Figure 3:**
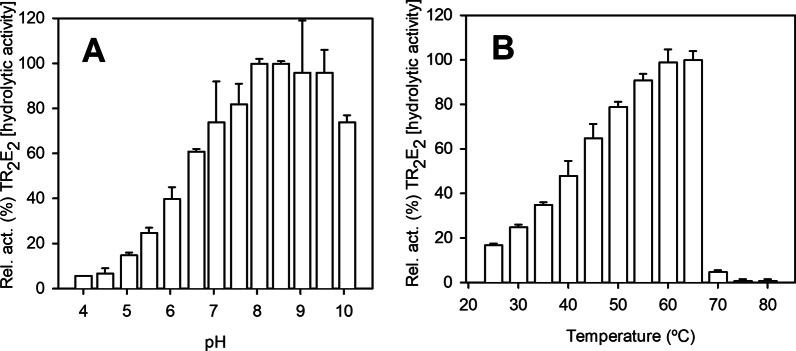
Optimal parameters for assessing the hydrolytic activity of purified TR_2_E_2_. A) TR_2_E_2_ activity toward 3‐OTfBE at different pH values. Reaction conditions: [protein]: 31 μM; [3‐OTfBE]: 7.2 mM; pH: 50 mM Britton–Robinson (BR) buffer with a pH ranging from 3 to 11; reaction volume: 100 μL; and *T*: 30 °C. B) TR_2_E_2_ activity toward 3‐OTfBE at different temperatures. Reaction conditions: [protein]: 31 μM; [3‐OTfBE]: 7.2 mM; *T*: 25–80 °C; pH: 100 mM K_2_HPO_4_ buffer, pH 7.5; and reaction volume: 100 μL. In both cases, the reactions were performed in triplicate and were incubated for 15 min at 750 rpm, after which 300 μL of a solution composed of acetonitrile, H_2_O and formic acid (10 : 10 : 0.6) were added to stop the reaction. Substrate depletion was detected using HPLC. The figure was created using SigmaPlot 14.0 software.

**Figure 4:**
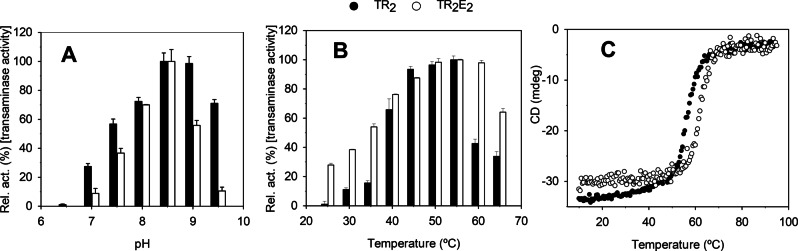
Optimal parameters for the transaminase activity and stability of purified TR_2_ (black) and TR_2_E_2_ (white). A) pH profiles of TR_2_ and TR_2_E_2_. Reaction conditions: [protein]: 31 μM; [hexanal]: 14 mM; [PLP]: 1 mM; [NPEA]: 14 mM; *T*: 40 °C; pH: 50 mM BR buffer with a pH ranging from 3 to 11; reaction volume: 200 μL. B) Thermal profiles of TR_2_ and TR_2_E_2_. Reaction conditions: [protein]: 31 μM; [hexanal]: 14 mM; [PLP]: 1 mM; [NPEA]: 14 mM; *T*: 22–80 °C; pH: 100 mM K_2_HPO_4_ buffer, pH 7.5; and reaction volume: 200 μL. C) The thermal denaturation curves of TR_2_ and TR_2_E_2_ at pH 7.0, as measured by changes in ellipticity at 220 nm. The figure was created using SigmaPlot 14.0 software.

Furthermore, we tested 3‐OTfBE as a substrate. This ester is used to synthesize precursors of gliptins, namely, β‐amino acids obtained through bienzymatic cascade reactions catalyzed by esterases and transaminases.[^22^, ^23^] TR_2_E_2_ was shown to efficiently hydrolyze 3‐OTfBE (*k*_cat_: 32.47±1.13 min^−1^). Using 3‐OTfBE and High Performance Liquid Chromatography (HPLC) determinations, TR_2_E_2_ showed maximal hydrolytic activity at pH ranging from 8.0 to 9.5 (Figure 3A) and temperatures ranging from 60 to 65 °C (Figure 3B). The optimal pH (ca. 8.5) and temperature (ca. 50–55 °C) for the transaminase activity of the variant TR_2_E_2_ were similar to those of TR_2_ itself (Figure 4A and B). The finding that both TR_2_ and TR_2_E_2_ retained high activity at 50–65 °C was consistent with their denaturation temperatures (56.23±0.09 °C and 61.85±0.11 °C, respectively), as revealed by circular dichroism spectroscopy (Figure 4C).

Diffracting crystals were obtained for TR_2_E_2_ as well. Datasets for each crystal were collected and refined to a resolution of 3.3 Å. All statistics for the data are presented in Table S1. The artificial esterase catalytic triad was observed in the TR_2_E_2_ crystal structures and is composed of Ser172 and His173 of chain A (Figure 1C, magenta) and Glu317 of chain B (Figure 1C, green); similar to the transaminase's active site, the esterase site is composed of a functional protein dimer. Figure 1B presents the surface of the TR_2_ protein (monomer A in light green and monomer B in wheat) compared to TR_2_E_2_ (monomer A in light purple and monomer B in blue) (Figure 1D). The position of the mutation used to gain the esterase function is shown in magenta and features good accessibility, as it is located at the surface, whereas the transaminase catalytic base is located within a small notch, as shown by the bound PLP (highlighted in ball and sticks in Figure 1D). The RMSD between TR_2_ and TR_2_E_2_ was calculated to be 0.71 Å between 299 of 389 C_α atoms of the aligned residues, indicating that no large conformational change occurs due to mutations, as also confirmed by the transaminase activity assay (Tables 1 and S4). Notably, the distance between both active sites is ≈20 Å, with the esterase site being more solvent‐exposed and located at the beginning of the tunnel and the transaminase site being more buried in the protein structure.

**Figure 5:**
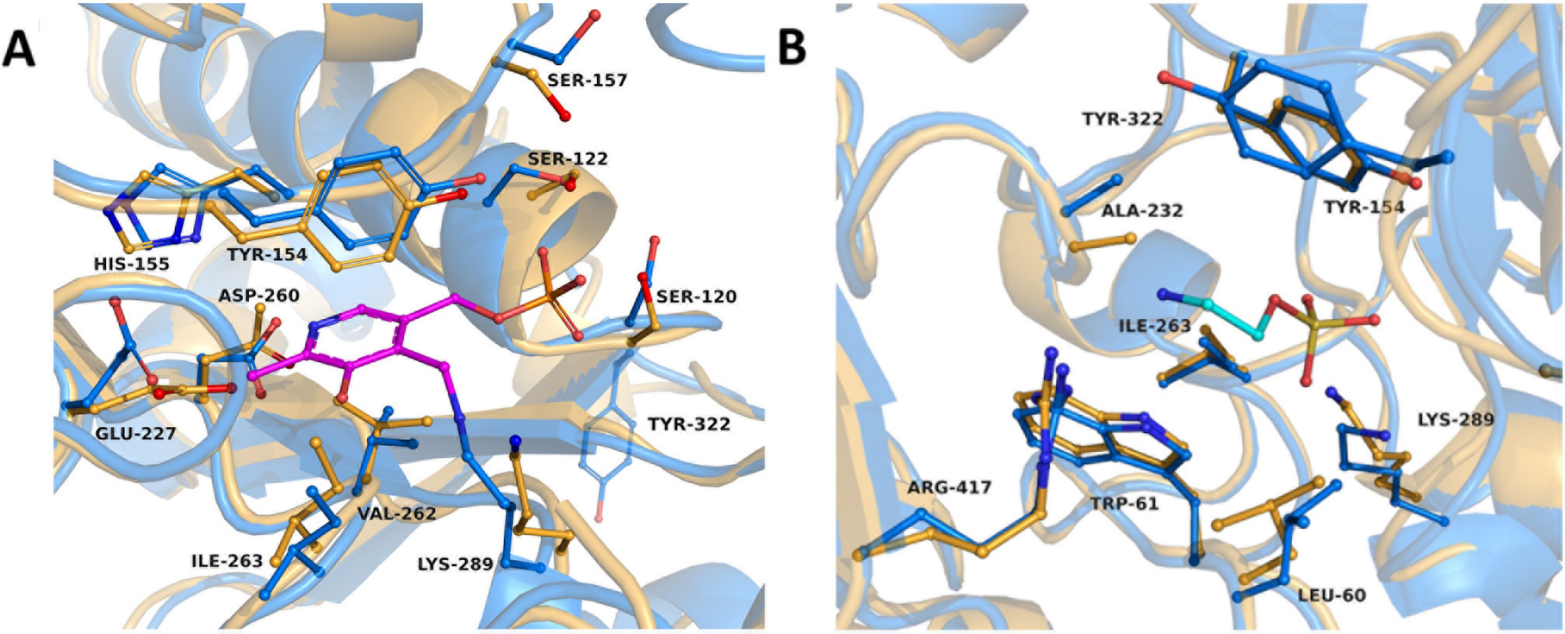
Comparison of the transaminase catalytic site in the TR_2_ and TR_2_E_2_ structures. A) Representation of the catalytic center of TR_2_E_2_ with PLP (magenta) bound to Lys289. The TR_2_ (orange) structure is overlaid with the TR_2_E_2_‐PLP bound structure (blue). B) Overlay of the EOS inhibitor binding site. The TR_2_ structure (orange) is compared with the TR_2_E_2_ structure (blue) within the EOS (cyan) binding site. The introduction of the mutation does not alter either the PLP or the EOS binding site. The figure was created using PyMOL Version 2.3.2 software.

Additionally, we succeeded in growing crystals and solving the structure of TR_2_E_2_ bound to the PLP cofactor (Figure 5A, resolution of 3.5 Å) and in complex with the ethanolamine O‐sulfate (EOS) inhibitor (Figure 5B, resolution of 3.6 Å), located in the transaminase catalytic center. All data statistics are presented in Table S1. The functional TR_2_E_2_ dimer shows one monomeric subunit (chain A) with PLP bound to the ϵ‐amino group of the catalytic base Lys289, forming an internal aldimine (Figure 5A). The other monomeric subunit (chain B) shows no linkage to the cofactor but an orientation in the catalytic center with stabilization of chain A, indicating that ω‐transaminase forms a functional dimer.

After the computational design of a hydrolase site in TR_2_ and characterization of the successful variant TR_2_E_2_, we wanted to test the ability of this validated *PluriZyme* to catalyze a cascade reaction of interest. Therefore, as a model reaction, we chose to synthesize 3‐ATfBA from 3‐OTfBE; this molecule is an intermediate of gliptins.[^22^, ^23^] Simulation of the reaction by a local PELE exploration showed efficient 3‐ATfBE and 3‐OTfBE catalytic (hydrolytic) binding poses: 23 catalytic events for 3‐ATfBE and 42 for 3‐OTfBE when the serine‐substrate distance threshold was set to 4 Å (Figure S12). Glyceryl tripropionate, an example of an ester commonly hydrolyzed by most esterases,^[36]^ had 121 catalytic events with an associated experimental *k*_cat_ of 388.1 min^−1^ (Table S5). Thus, we expected that 3‐OTfBE would be preferentially hydrolyzed. This prediction was confirmed experimentally by showing that the catalytic efficiency of TR_2_E_2_ for the hydrolysis of 3‐OTfBE was 480‐fold higher than that of 3‐ATfBE (Table 1).

**Figure 6:**
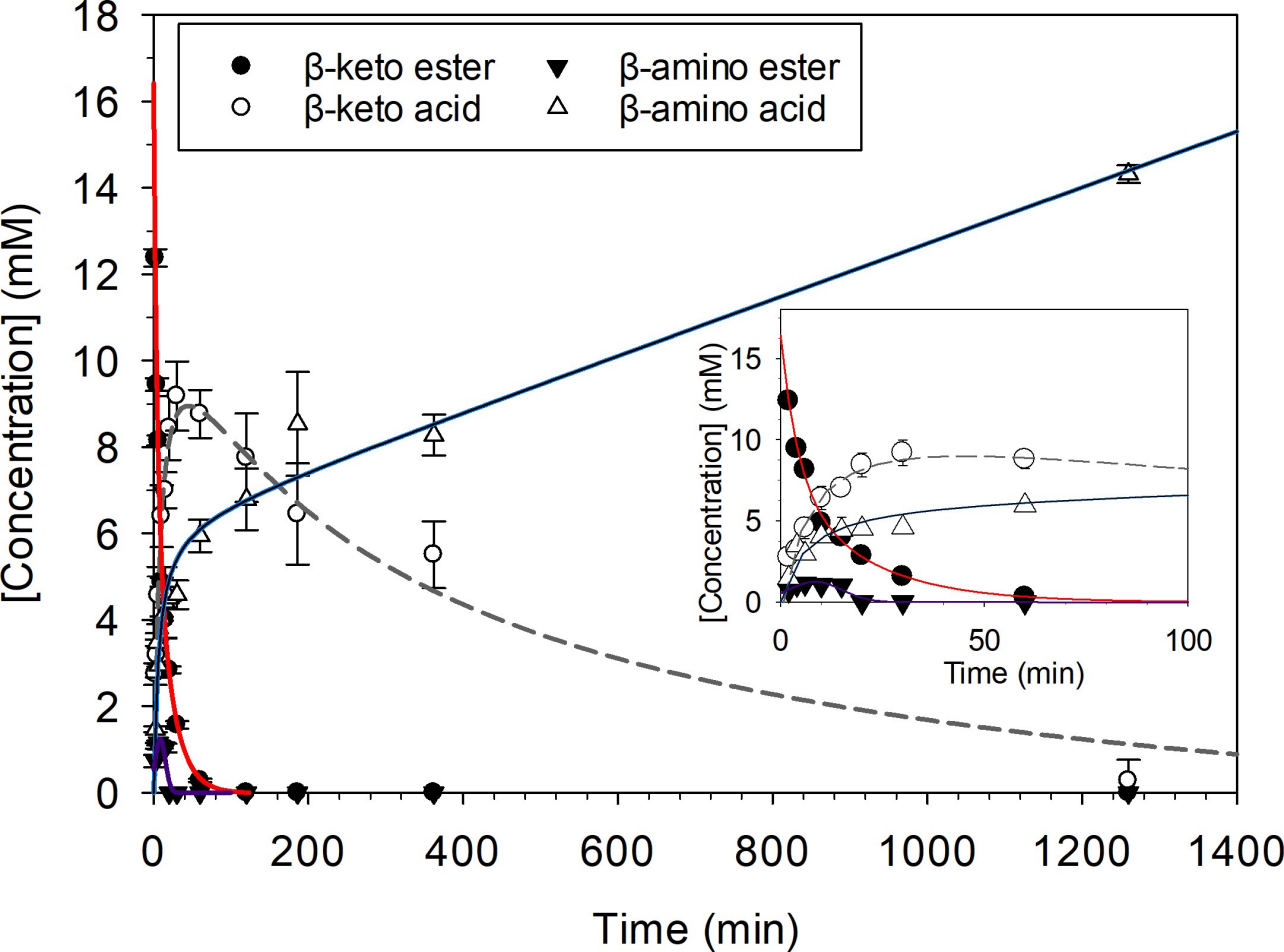
Concentrations of substrates, intermediates and the final product obtained for the conversion of 3‐OTfBE by the *PluriZyme* TR_2_E_2_. The following reaction conditions were used: [protein]: 31 μM; [3‐OTfBE]: 14 mM; [PLP]: 1 m; [NPEA]: 14 mM; pH: 100 mM K_2_HPO_4_ buffer, pH 7.5; reaction volume: 100 μL; and *T*: 40 °C. The figure was created using SigmaPlot 14.0 software.

We set up a one‐pot reaction at 40 °C and pH 7.5 with all the reagents necessary for the hydrolysis and transamination reactions, i.e., 3‐OTfBE, PLP (as a cofactor) and NPEA (as amine donor). After adding the TR_2_E_2_ *PluriZyme*, the levels of the substrate 3‐OTfBE, the intermediates 3‐ATfBE and 3‐OTfBA, and the product 3‐ATfBA were quantified using HPLC over 21 h (Figure 6 and Figure S13), and the e.e. was analyzed using Gas Chromatography (GC). The same experimental conditions were employed with TR_2_ and a control reaction with no enzymes.

We found that the β‐keto ester substrate 3‐OTfBE (concentration, 14 mM) was fully converted (>99 %) after 60 min, and that the reaction proceeded via the formation of the β‐keto acid 3‐OTfBA (initial rate: 0.702±0.07 mM min^−1^), which reached the maximal concentration (9.2±0.8 mM) at 30 min. Afterward, the β‐amino acid (*R*)‐3‐ATfBA (initial rate: 0.479±0.08 mM min^−1^) was finally produced (14.3±0.21 mM; e.e. >99 %). The intermediate β‐amino ester 3‐ATfBE was a minor product of the reaction during the early stages of the reaction (max. conc. 1.05±0.03 mM at 15 min; initial rate: 0.290±0.04 mM min^−1^), but was subsequently consumed. This result is consistent with the higher transamination efficiency (ca. 200‐fold) of TR_2_E_2_ for 3‐OTfBA compared to 3‐OTfBE and the 480‐fold lower capacity of TR_2_E_2_ to hydrolyze 3‐ATfBE compared to the initial substrate 3‐OTfBE (Table 1). Notably, when the reaction was established in the presence of TR_2_, no β‐amino acid 3‐ATfBA was formed, and only a minor conversion to 3‐ATfBE β‐amino ester was detected (Figure S14).

**Scheme 2:**
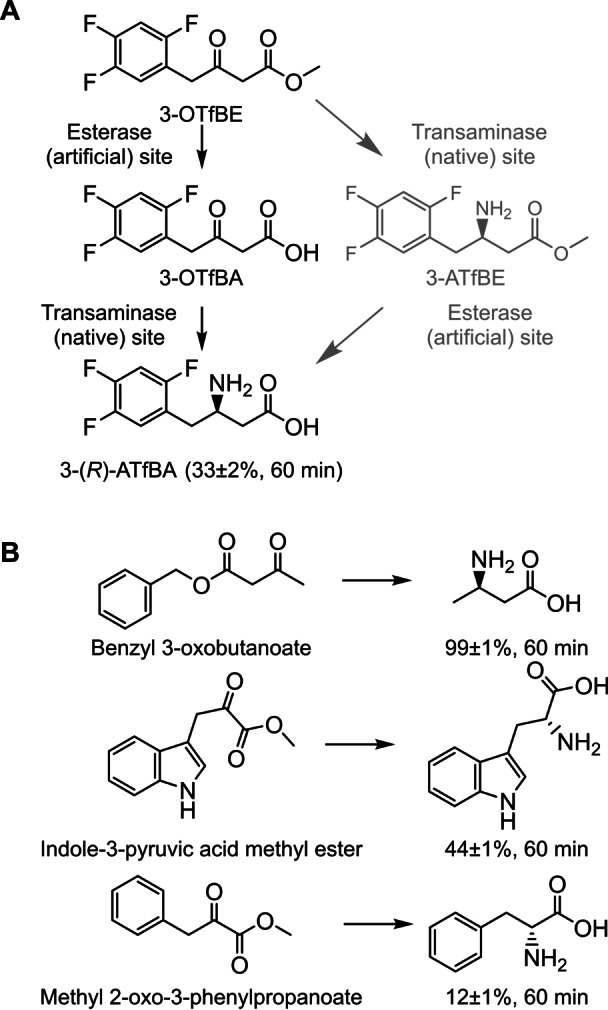
Schematic representation the one‐pot reaction products for converting a set of β‐keto esters by TR_2_E_2_. A) Schematic representation of the two possible routes for converting 3‐OTfBE into 3‐ATfBA. As shown in the figure, the preferential route is the production of 3‐ATfBA via hydrolysis of 3‐OTfBE to 3‐OTfBA, which then yields (*R*)‐3‐ATfBA, and not via formation of 3‐ATfBE (gray color). B) Schematic representation of one‐pot synthesis of amino acids from a set of β‐keto esters. The conversion, determined by HPLC for all substrates, at 60 min is shown. The figure was created using ChemDraw 18.2.

In summary, of the two possible routes by which the β‐keto ester substrate may yield the β‐amino acid in the presence of TR_2_E_2_, we identified the preferential route as the conversion of the β‐keto ester to the β‐keto acid at the hydrolytic site, followed by its subsequent amination at the transaminase site (Scheme 2A).

In order to demonstrate the catalytic potential of the dual‐function TR_2_E_2_ enzyme, a range of additional substrates were further tested. A series of β‐keto esters were tested as potential substrates, because they are commercially available, and have various potential applications as biomedical compounds or are important synthons for natural products synthesis. Both hydrolase and transaminase activities were evaluated and quantified.

In detail, a set of 7 β‐keto esters were tested, which included the aromatic substrates benzyl 3‐oxobutanoate, indole‐3‐pyruvic acid methyl ester, methyl 2‐oxo‐3‐phenylpropanoate, 2‐methyl‐4‐oxo‐4*H*‐pyran‐3‐yl propionate, ethyl (4‐ethoxyphenyl)(oxo)acetate, and ethyl (1‐hydroxy‐4‐oxocyclohexyl)acetate, and the linear alkyl β‐keto ester dimethyl 2‐oxoglutarate (Table S6; Figure S11). Among them, 6 were hydrolyzed by the biotic artificial esterase site in TR_2_E_2_, at different rates. In addition, 6 were also accepted as amine acceptors in transaminase reaction by the biotic native transaminase site in TR_2_E_2_; they were converted at a lower rate compared to hexanal.

We first set up a one‐pot cascade reaction with benzyl 3‐oxobutanoate, using similar conditions as those used for 3‐OTfBE (see Figure 6), as it was one of the preferred β‐keto esters for both hydrolase and transaminase reactions from those seven tested, and the possible end β‐amino acid product of the reaction is an important intermediate for the production of biomedicals.^[36]^ The products were quantified using HPLC. We found that the β‐keto ester benzyl 3‐oxobutanoate (concentration, 14 mM) was converted to a high extent (>95 % after 20 min and 99 % after 60 min; Figure S15). The reaction proceeded via the rapid formation of benzyl alcohol (initial rate: 5.85±0.04 mM min^−1^) and 3‐oxo‐butyric acid, finally yielding the expected amine‐product (*R*‐3‐aminobutyric acid, e.e. >99 %) (Scheme 2B), as confirmed by high‐resolution mass spectrometry (HR‐MS; Figure S16) analysis which determined the *m*/*z* ratio 104.0701 for the composition C_4_H_10_NO_2_ ([*M*+H]) with an error of less than 5 ppm with respect to the theoretically calculated mass for that composition.

Under similar conditions and by HPLC, we further succeed in the one‐pot conversion at 60 min of the aromatic β‐keto ester indole‐3‐pyruvic acid methyl ester to tryptophan (44±1 %, 60 min; Figure S17) and of methyl 2‐oxo‐3‐phenylpropanoate to phenylalanine (12±1 %, 60 min; Figure S18) (Scheme 2B).

Structural analysis revealed a 20 Å distance between both active sites, with the esterase site being solvent‐exposed and the transaminase site buried in the protein structure (Figure 1D), thus ensuring that the transfer of the reaction intermediate within the protein structure is theoretically possible. Actually, we tested the migration of 3‐OtfBA using PELE from the esterase site to the transaminase one for TR_2_E_2_ and for two mutants, TR_2_E_2_A232F/L60F and TR_2_E_2_A232F/F89W, designed to close the access channel to the transaminase site. As it can be seen in the energetic profiles of those simulations, the substrate can easily reach the transaminase site from the esterase site in TR_2_E_2_ but not in the two mutants (Figure S19). The feasibility of such a connection between sites was further proven by experimentally characterizing the TR_2_E_2_A232F/L60F. Indeed, this mutant retained the affinity and hydrolytic activity for 3‐OTfBA (*K*_m_ of 2.063±0.046 mM and *k*_cat_ of 28.89±1.52 min^−1^), while showing lower affinity and transamination activity for 3‐OTfBA (*K*_m_ of 9.98±0.32 mM and *k*_cat_ of 44.87±4.14 min^−1^) (Figure S20), compared to TR_2_E_2_ (see Table 1). This indicates that mutations affect the substrate access to the transaminase site but not the esterase site. This was further validated by following the one‐pot conversion from 3‐OTfBE to 3‐(*R*)‐ATfBA by HPLC (Figure S21), which was 3‐fold times lower in the mutant at a short incubation time (6 minutes); however, at 60 min an almost full conversion was achieved with TR_2_E_2_ and TR_2_E_2_A232F/L60F, most likely due to the possibility that the intermediate diffuse from one side to the other at longer incubation times.

## Conclusion

We have documented the successful design and construction of a *PluriZyme* integrating biotic transaminase and esterase activities and its application in a cascade reaction. We achieved these results by applying computational techniques to a class III ω‐TA and by examining a set of 20 mutants supporting presumptive catalytic triads and oxyanion holes for ester hydrolysis. Of the six variants retaining transaminase activity, one acquired additional esterase activity and was capable of hydrolyzing multiple esters at turnover numbers approaching those of highly efficient similar native esterases/lipases reported in the literature.^[37]^ Interestingly, TR_2_E_2_ was designed using a site where a loop was missing in the crystal structure of the original transaminase TR_2_; the loop contained one mutation and was modeled with Prime and refined through MD simulations (Figure S22). Thus, the insertion of the hydrolytic site helped stabilize this region by adding two internal hydrogen bonds, improving the thermostability of the enzyme. The incorporation of a second biological activity to support a cascade reaction into an enzyme may not occur at the expense of the activity and stability of the original enzyme.

We further revealed that this bioinspired design facilitates the one‐step synthesis of a sitagliptin intermediate, specifically the conversion of 3‐OTfBE into 3‐ATfBA in a single one‐pot reaction (Scheme 2). This β‐amino acid is commonly synthesized through a bienzymatic cascade reaction that involves an esterase and a transaminase or a recombinant *Escherichia coli* strain coexpressing both enzymes.[^22^, ^23^, ^38^] A series of chiral 3‐substituted cyclo‐hexylamine derivatives, capsaicin analogs and nylon‐6 monomer 6‐aminohexanoic acid, to cite some significant examples, have also been synthesized by combining hydrolases with ωTAs as separate catalytic entities.[^39^, ^40^, ^41^] It is worth mentioning that to date, only three native hydrolases, namely, those from *Candida rugosa*, *Pseudomonas avellanae* and *P. stutzeri*,[^22^, ^23^, ^37^] have been reported as capable of hydrolyzing 3‐OTfBE and used in the cascade synthesis of 3‐ATfBA. Thus, the computational approach used here expands the range of biocatalysts supporting this transformation. The TR_2_E_2_ *PluriZyme* achieved (*R*)‐3‐ATfBA production rates (ca. 0.479±0.08 mM min^−1^) in the range of the previously reported bienzymatic cascade systems (ca. 0.05–0.4 mM min^−1^).[^22^, ^38^] Having said that, further studies are needed to determine whether using a single artificial enzyme with two “bioactive” sites in cascade reactions, is more efficient than a conventional multienzyme system and natural or artificial chimeric enzymes with hybrid activities.[^11^, ^12^, ^13^] Among the advantages, it is worth mentioning that using one enzyme with two biotic sites catalysing different chemistry would reduce the development and costs associated with the production of several enzymes, each supporting one chemistry, or favoring reaction yields by helping the substrates channeling; however, these and other advantages, yet to be defined, may vary from enzyme to enzyme.

In this study, we used a transaminase as a scaffold to introduce esterase activity. We consider this workflow more appropriate than the other approach, namely, exploring esterases or lipases to find alternative binding sites to accommodate a new site supporting transaminase activity. This design would require cofactor binding sites or large channels to accommodate the substrates, which may be technically more challenging.

The bioinspired design of the *PluriZyme* described here clearly provides a computational and experimental framework to develop future enzyme designs integrating two biotic‐catalytic centers, which, being independent, can also work in synergy for cascade reactions. In this direction, the fact that TR_2_E_2_ could convert multiple keto esters exemplified the catalytic potential of such designs. An important aspect of such future work will be the configuration of *PluriZymes* that, by supporting multiple biological activities through the assembly of appropriated binding and catalytic pockets, might reassemble in vitro “artificial metabolisms” as pathways for the preparation of valuable biomolecules, including medicines.

We would like to highlight that biosynthetic enzymes with different domains, in which a substrate is channelled between two biotic sites catalysing different chemistries, already exist in Nature.^[13]^ Therefore, the dual active site strategy herein designed, namely the artificial TR_2_E_2_ enzyme, may represent a complementary one to that already employed by Nature for multi‐domain enzymes. In addition to that, the catalytic promiscuous character of the enzyme design herein reported may complement the catalytic potential of engineered promiscuous enzymes that are known to represent attractive alternatives to conventional chemical catalysts. A recent example is the promiscuous acyltransferase activity of certain hydrolases that can catalyze not only the formation of esters, but also the formation of amides, carbonates, and carbamates in water.^[42]^ While such catalytic promiscuous enzymes can potentially perform two different chemistries, their use in cascade reactions have been rarely investigated compared to our design.

## Conflict of interest

The authors declare no conflict of interest.

1

## Data Availability

The data that support the findings of this study are available in the Supporting Information of this article.
